# A sensitive and reproducible qRT-PCR assay detects physiological relevant trace levels of *FMR1* mRNA in individuals with Fragile X syndrome

**DOI:** 10.1038/s41598-023-29786-4

**Published:** 2023-03-07

**Authors:** Devan Straub, Lauren M. Schmitt, Anna E. Boggs, Paul S. Horn, Kelli C. Dominick, Christina Gross, Craig A. Erickson

**Affiliations:** 1 Division of Child and Adolescent Psychiatry, Cincinnati Children’s Hospital Medical Center, 3333 Burnet Ave., Cincinnati, OH 45229-3039 USA; 2 Division of Behavioral Medicine and Clinical Psychology, Cincinnati Children’s Hospital Medical Center, 3333 Burnet Ave., Cincinnati, OH 45229-3039 USA; 3 Department of Pediatrics, University of Cincinnati College of Medicine, 3333 Burnet Ave., Cincinnati, OH 45229-3039 USA; 4 Department of Psychiatry and Behavioral Neuroscience, University of Cincinnati College of Medicine, Stetson Building Suite 3200, 260 Stetson Street, Cincinnati, OH 45267-0559 USA; 5 Division of Neurology, Cincinnati Children’s Hospital Medical Center, 3333 Burnet Ave, Cincinnati, OH 45229-3039 USA

**Keywords:** Neuroscience, Diseases of the nervous system

## Abstract

Fragile X syndrome (FXS) is the most common inherited intellectual disability. FXS is caused by a trinucleotide repeat expansion in the 5′ untranslated region of the *FMR1* gene, which leads to gene methylation, transcriptional silencing, and lack of expression of Fragile X Messenger Riboprotein (FMRP). Currently available FXS therapies are inefficient, and the disease severity is highly variable, making it difficult to predict disease trajectory and treatment response. We and others have recently shown that a subset of full-mutation, fully-methylated (FM–FM) males with FXS express low amounts of FMRP which could contribute to phenotypic variability. To better understand the underlying mechanisms, we developed a sensitive qRT-PCR assay to detect *FMR1* mRNA in blood. This assay reproducibly detects trace amounts of *FMR1* mRNA in a subset of FM–FM males, suggesting that current Southern Blot and PCR determination of FM–FM status is not always associated with complete transcriptional silencing. The functional relevance of trace-level *FMR1* mRNA is confirmed by showing a positive correlation with cognitive function; however, phenotypic variability is not fully explained by *FMR1* expression. These results corroborate the need for better molecular assays for FXS diagnosis and encourage studies to elucidate the factors contributing to the phenotypic variability of FXS.

## Introduction

Fragile X Syndrome (FXS) is the most frequent inherited form of intellectual disability and the most common monogenic form of autism spectrum disorder^1,2^. Although the genetic cause of FXS, a CGG trinucleotide repeat expansion in the 5′ untranslated region (UTR) of the Fragile X Messenger Riboprotein gene 1 (*FMR1*) leading to loss of the Fragile X Messenger Riboprotein (FMRP), was discovered more than two decades ago, many aspects of the disorder are still incompletely understood^3^. Therefore, current treatments are mainly symptomatic with limited efficacy for core developmental impairments associated with the disorder^4^.

The CGG repeat expansion in the *FMR1* gene is usually categorized into “normal” (≤ 44 repeats), intermediate or grey zone (45–54), pre-mutation (55–200 repeats) and full mutation (> 200 repeats)^2,5^. While premutation carriers can develop health issues such as Fragile X-associated tremor/ataxia syndrome (FXTAS), most commonly in men, and Fragile X-associated primary ovarian insufficiency (FXPOI) in women^6^, in general, only individuals with a full mutation have gene hypermethylation, subsequent reduction in FMRP expression and FXS.

Even though FXS is a monogenic disorder, FXS is marked by considerable phenotypic variability^7^. Some affected individuals can obtain high school and associate degrees, while others may remain nonverbal into adulthood. This variability is partially caused by the location of the *FMR1* gene on the X chromosome, as phenotypic presentation in females with one fully expanded allele can significantly vary due to random X chromosome inactivation^8,9^. However, even males with FXS have a wide range of phenotypes suggesting additional contributing factors^7^.

As a first hint towards the potential contributing mechanisms, we and others showed that in a fraction of full mutation/fully methylated (FM–FM) male individuals, low levels of FMRP can be detected in peripheral blood^10–16^. Further analyses revealed that FMRP levels are positively associated with IQ across both males and females with FXS suggesting that the residual expression of FMRP is physiologically and functionally meaningful^9,10,13–15^. Notably, lower levels of FMRP were shown to associate with an autism diagnosis in individuals with FXS^13^, and overall, intellectual disability is more severe in individuals with lower FMRP levels even when parental IQ was considered^14^.

Mechanistically, these results imply that the *FMR1* gene is transcribed even in individuals with a repeat expansion larger than 200. Indeed, previous studies show that patients with full mutation FXS can express low levels of *FMR1* mRNA^17^, suggesting that hypermethylation may be incomplete^18^; however, the extent to which transcription occurs has not been analyzed in a systematically validated and reproducible assay. The CGG expansion in the 5′UTR of the mRNA is expected to reduce mRNA translation and could even produce a toxic gain-of-function effect as seen in premutation carriers leading to FXTAS- or FXPOI-related symptoms and neurodegeneration^19,20^. In line with this hypothesis, increased mRNA levels in individuals with full mutation FXS have been associated with increased irritability scores^21^. A better understanding of the mechanisms and implications of low level FMRP expression in FM–FM FXS and how it is related to *FMR1* gene transcription is needed to enable improved and personalized treatment strategies based on an individual’s predicted disease trajectory.

To this end, we developed a highly sensitive and reproducible quantitative real-time polymerase chain reaction (qRT-PCR)-based method to reliably detect low amounts of *FMR1* mRNA in peripheral whole blood. We use this assay to assess if *FMR1* mRNA can be reproducibly detected in FM–FM males with FXS, to evaluate the relationship between *FMR1* mRNA and FMRP expression, and to establish initial clinical utility of the assay by correlating *FMR1* mRNA with IQ. In the future, this assay could be an important part of the molecular diagnosis of FXS, may be useful in predicting clinical outcomes, and help developing personalized and improved therapeutic strategies.

## Results

### The optimized qRT-PCR assay for *FMR1* mRNA quantification is highly reproducible

A human *FMR1* mRNA-specific qRT-PCR assay was optimized to quantify *FMR1* mRNA in whole blood from individuals with FXS and typically developing controls (TDCs) (see “Methods” section for details). A total of 27 PCR assay plates (96 wells) were used for this analysis. One-hundred thirty-eight participants (148 samples) were ran on 22 plates (Table 1, see “Methods” section) and the other five plates were used as test plates. These plates passed the following criteria: (1) a standard curve with at least two of three replicates that have a Ct standard deviation below 0.5 and pass the Grubb’s test, (2) an efficiency percentage between 90 and 100%, and (3) an R^2^ value greater than 0.980. Data of the overall analyses for these plates are shown in Table 2.

**Table 1:** Demographic information.

	N	Age			*FMR1* concentration (pM)			
		Average	SD	Range	Average	Median	SD	Range
Male	83	22.0	16.3	2.0–67.6	5.66E−09	3.77E−09	5.39E−09	0.0 to 1.83E−08
Typically developing controls	26	30.8	17.4	5.6–63.8	1.07E−08	1.02E−08	2.95E−09	6.43E−09 to 1.83E−08
Premutation carriers	2	58.0	13.5	48.5–67.6	1.63E−08	1.63E−08	8.11E−11	9.44E−09 to 1.25E−08
Fragile X syndrome	55	16.5	12.1	2.0–48.6	2.67E−09	1.07E−09	3.76E−09	0.0 to 1.31E−08
FXS—nonmosaic	20	17.9	14.4	4.5–67.6	5.72E−10	0.00E + 00	1.06E−09	0.0 to 4.33E−09
FXS—repeat mosaic	9	38.4	48.2	2.0–36.7	4.69E−09	1.49E−09	5.22E−09	3.42E−10 to 1.31E−08
FXS—methylation mosaic	1	43.0	N/A	43	1.02E−08	1.02E−08	N/A	1.02E−08
FXS—repeat/methylation mosaic	7	14.5	11.5	5.0–36.3	5.64E−09	3.77E−09	3.26E−09	2.80E−09 to 1.13E−08
Female	55	33.6	18.9	1.2–66.2	1.07E−08	9.94E−09	5.10E−09	7.99E−10 to 2.52E−08
Typically developing controls	6	29.7	26.6	3.6–66.2	8.79E−09	7.86E−09	2.53E−09	6.85E−09 to 1.37E−08
Premutation carriers	27	44.0	11.3	15.0–62.3	1.37E−08	1.25E−08	4.96E−09	5.37E−09 to 2.52E−08
Fragile X syndrome	22	21.9	17.5	1.2–61.3	7.47E−09	7.20E−09	3.38E−09	7.99E−10 to 1.33E−08
FXS—Nonmosaic	11	17.6	10.6	3.5–62.3	6.92E−09	6.84E−09	3.34E−09	1.47E−09 to 1.33E−08
FXS—repeat mosaic	3	36.4	27.4	7.1–61.3	9.22E−09	9.94E−09	2.13E−09	6.82E−09 to 1.09E−08
FXS—methylation mosaic	1	3.3	N/A	3.2	4.81E−09	4.81E−09	N/A	4.81E−09
FXS—repeat/methylation mosaic	3	29.6	29.6	1.2–60.3	6.39E−09	6.37E−09	5.60E−09	7.99E−10 to 1.20E−08

*FMR1* Fragile X Messenger Riboprotein 1, *SD* Standard Deviation.

**Table 2:** qRT-PCR plate analysis.

Criteria	Average	Range
Standard points removed per plate	0.481	0–4
Average R^2^	0.999	0.997–1
Average efficiency, %	95.740	91.954–99.042

Intra-assay variability, or the variability between replicates, was measured by averaging the coefficient of variation (CV) for 143 sets of quintuplicates. The 19 participants that were either removed from analysis due to insufficient assay quality (see below, 4 participants) or had their *FMR1* mRNA concentrations set to zero (15 participants) were excluded from this calculation. Our intra-assay variability was 5.02% (Table 3).

**Table 3:** Overall variability of the *FMR1* mRNA assay.

Overall assay variability	Sample size	%CV ± SD	
Intra-assay (119 participants)	143 (quintuplicates)	5.02 ± 2.61	
Inter-plate (14 participants)	28 (quintuplicates)	14.20 ± 11.16	
Inter-draw (8 participants)	25 (quintuplicates)	25.40 ± 16.17	

Variability for one participant	Sample Size	Mean ± SD	%CV
Inter-plate (TDC #1)	15 (quintuplicates)	8.87E−09 ± 1.61E−09	18.21
Inter-draw, same day (TDC #1, visit 1)	28 (quintuplicates)	9.95E−09 ± 1.62	16.3
Inter-draw, different days (TDC #1, visit 1 and 2)	4 (quintuplicates)	7.31E−09 ± 6.25E−10	8.55

*CV* Coefficient of variation, *SD* standard deviation.

Inter-assay and inter-draw variability were determined using 14 participants across the entire spectrum of *FMR1* expression and were analyzed in more detail for one typically developing control (TDC #1) (Table 3). The overall inter-assay variability was calculated by averaging the CVs of the 20% trimmed means of 14 participants for whom the same RNA sample was run on two different plates and was 14.20%. For the four FM–FM male participants the CV was 28.97%. The average CV of the other 10 participants in this group was 8.30%. This is expected as variability increases towards the lower detection limit. The inter-assay variability across 15 plates for one RNA tube from TDC #1 was 18.21%. Note that the inter-assay variabilities for two additional RNA tubes from that same draw had a CV of 11.21% (across 3 plates) and 12.94% (across 10 plates), and the mean variability for all three tubes was 14.12%.

The variability between different draws, or inter-draw variability, was assessed for draws on the same or on different days. For inter-draw variability on the same day, three tubes from TDC #1 drawn on the same day were used. The CV for the 20% trimmed means averaged over all assays from the three different RNA tubes drawn on the same day determined the same-day inter-draw variability and was 16.30%. For inter-draw variability on different days, TDC #1 and eight participants (3 FM–FM males, 2 mosaic males, 2 FXS females, 1 TDC female) with multiple draws were used. The inter-draw variability for TDC #1 was determined by calculating the CV between one tube drawn on visit 1 and one tube drawn on visit 2. The time between the two draws was 176 days. Two aliquots for each tube were run on the same plate and each aliquot’s 20% trimmed mean value was averaged together to give one value for each tube. The CV was 8.55% (Table 3). The inter-draw variability for the eight participants with multiple draws was calculated by averaging the CV between the 20% trimmed means for each draw (between 2 and 3) and was 25.40% (age at first draw (mean+/−SD): 11.3 ± 8.7 years, range 5–31 years). Note that six of the eight participants were between 5 and 10 years old at the time of the first blood draw and that the interval between the draws was 309 +/−228 days. This suggests that the different draws occurred over a time with large developmental changes for a majority of participants, which might have contributed to the higher variability.

### Freeze–thaw effect on *FMR1* mRNA concentration

**Figure 1:**
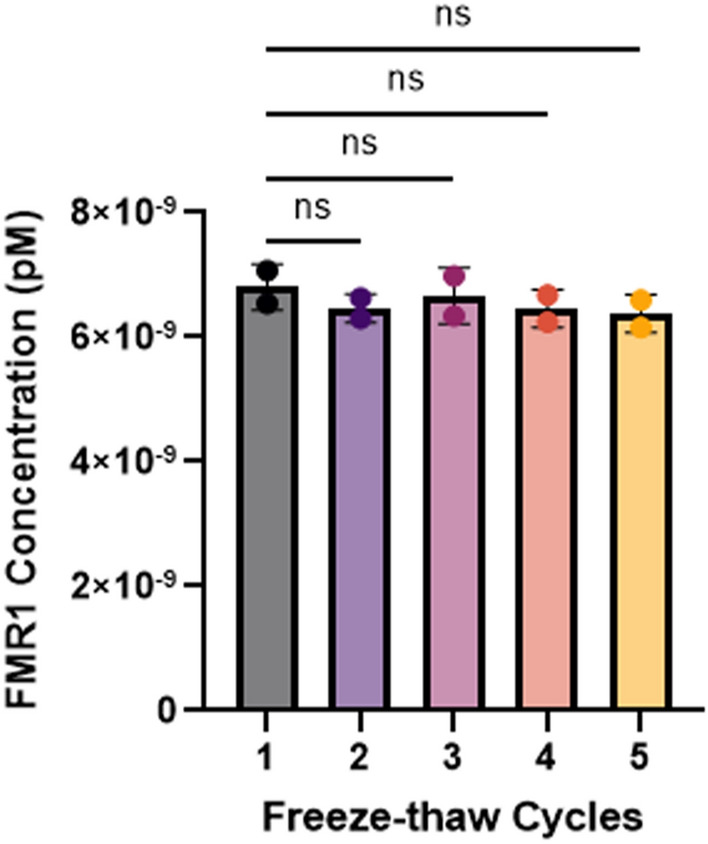
*FMR1* mRNA concentration is not significantly affected by multiple freeze–thaw cycles. Scatter dots indicate the 20% trimmed mean values for the 2 tubes per condition. There was no significant difference in *FMR1* mRNA concentration between any of the groups (Kruskal–Wallis test, *p* = 0.8) and no difference between the first freeze–thaw and the second (*p* = 0.4090), third (*p* = 0.7412), fourth (*p* = 0.4090), and fifth freeze–thaw cycle (*p* = 0.1864). P values for comparisons between freeze–thaw cycles are FDR (Benjamini and Hochberg)-corrected. *FMR1* Fragile X Messenger Riboprotein 1, *ns* Not Significant.

In addition, we evaluated the effect of multiple freeze–thaw cycles on *FMR1* mRNA concentration. The cycles ranged from 1 to 5 with 2 tubes per condition. There was no significant difference between the first freeze–thaw cycle and freeze–thaw cycles 2–5 (Fig. 1).

### *FMR1* mRNA levels are significantly different between FXS diagnostic groups

**Figure 2:**
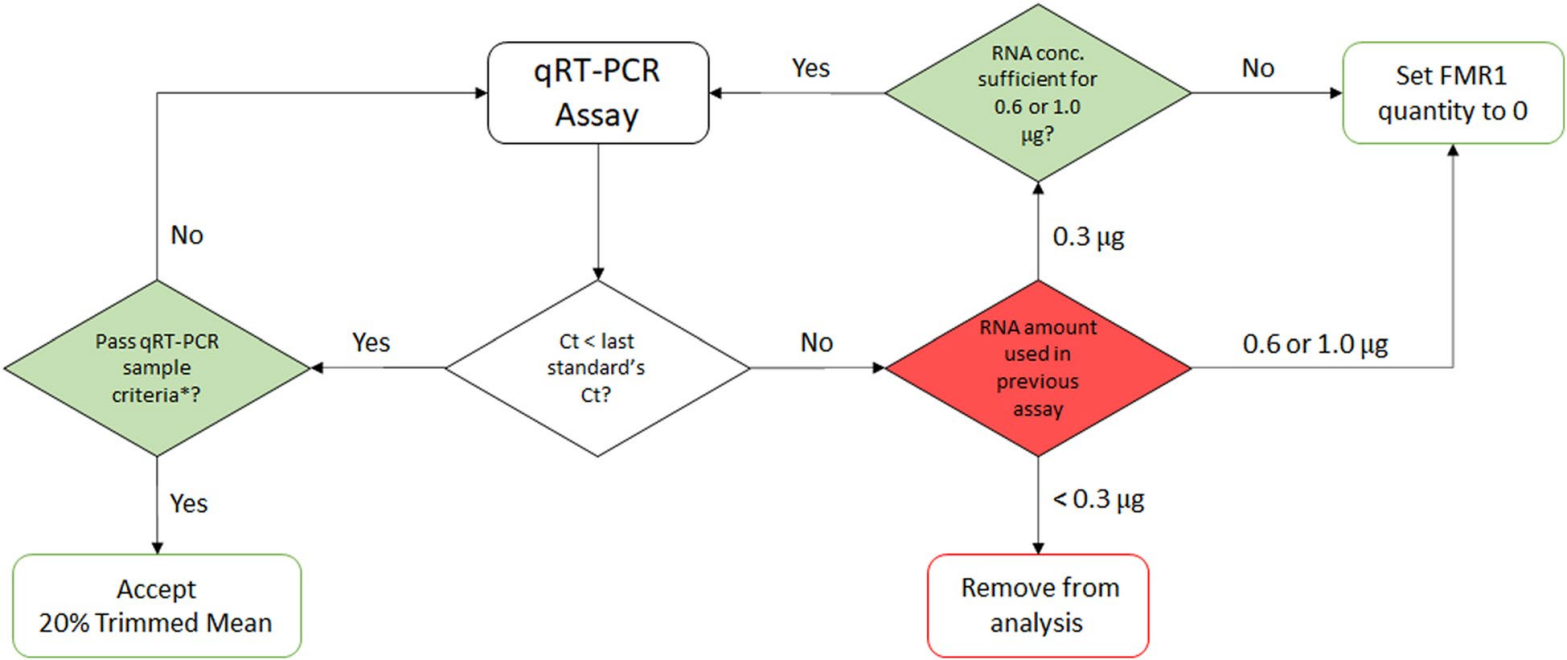
Sample acceptance criteria. *qRT-PCR sample criteria include a set of quintuplicates with a CV < 15%, no outliers, and no undetermined Ct values. *Ct* Cycle Threshold, *FMR1* Fragile X X Messenger Riboprotein 1.

*FMR1* mRNA was analyzed for 138 participants (148 samples) recruited from three diagnostic groups (FXS, PMC, and TDC). Based on the strategy shown in Fig. 2 and described in the “Methods” section, *FMR1* mRNA concentrations for 15 FXS participants were set to zero and four participants were removed from the analysis. For the eight participants with multiple draws and the 14 participants with two accepted runs from the same RNA tube, the 20% trimmed mean values of each accepted run were averaged.

**Figure 3:**
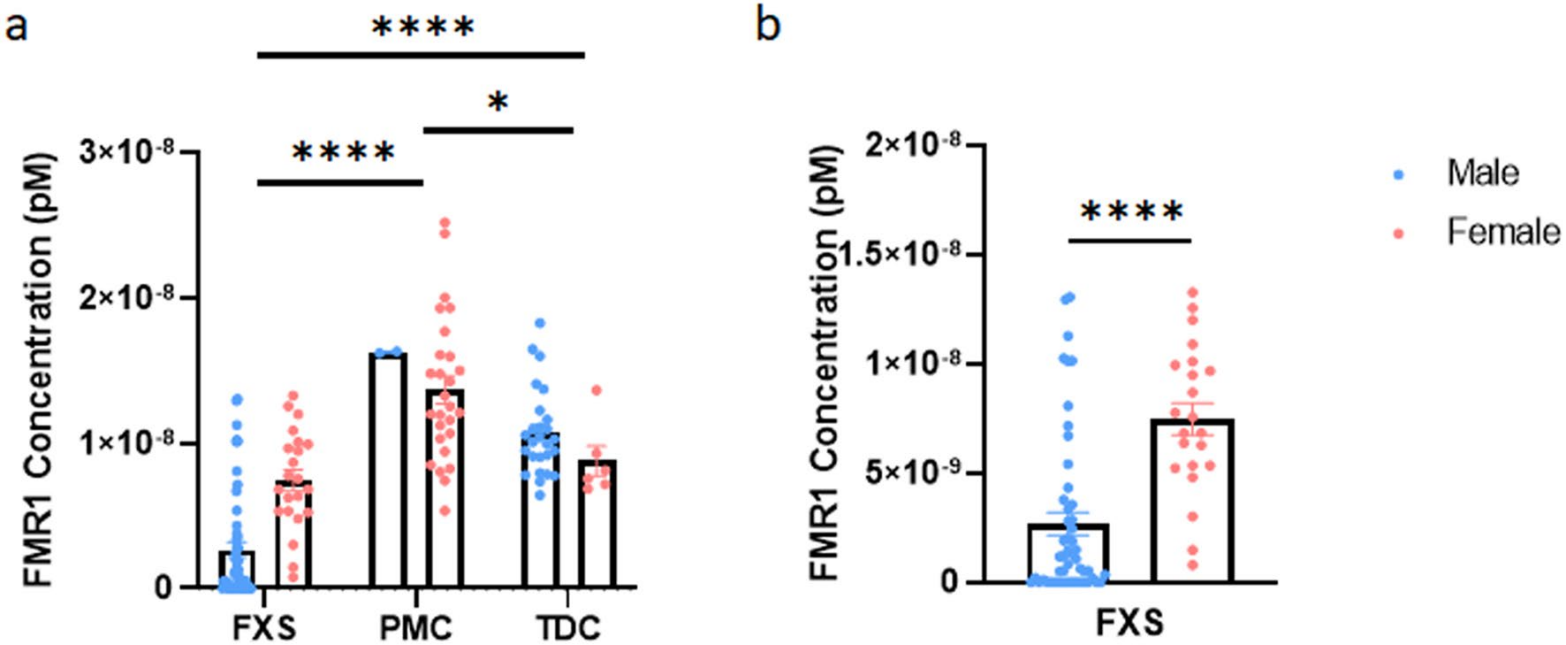
*FMR1* levels are reduced in participants with FXS. **a** *FMR1* mRNA concentration for all diagnostic categories. Male and females were grouped separately by diagnosis in the graphs, but each diagnostic category was grouped together regardless of sex for statistical analysis. Overall, there is a significant difference between diagnoses (Kruskal–Wallis test *p* < 0.0001). Posthoc test with the original FDR method of Benjamini and Hochberg reveal that participants with FXS have a significantly lower *FMR1* concentration than PMCs (*p* < 0.0001) and TDCs (*p* < 0.0001), while PMCs produce significantly higher *FMR1* mRNA levels than TDCs (*p* = 0.0450). **b** *FMR1* mRNA levels are significantly lower in males with FXS than females with FXS (two-tailed Mann–Whitney test U = 188, n_1_ = 51 n_2_ = 22, *p* < 0.0001). No significant difference is observed between male and female PMCs (two-tailed Mann–Whitney test, U = 12, n_1_ = 2 n_2_ = 27, *p* = 0.2414) or TDCs (two-tailed Mann–Whitney test, U = 40, n_1_ = 26 n_2_ = 6, *p* = 0.0695) (*not shown*). Error bars represent SEM. *FMR1* Fragile X Messenger Ribonucleoprotein 1, *FXS* Fragile X Syndrome, *PMC* Premutation Carriers, *TDC* Typically Developing Controls, *SEM* Standard Error of the Mean, *ns* Not Significant, ****Indicates *p* < 0.0001, *Indicates *p* < 0.05.

First, we examined *FMR1* mRNA concentrations among the different diagnostic groups. As expected, *FMR1* mRNA levels in participants with FXS were significantly lower than both PMCs and TDCs. In contrast, PMCs had significantly higher *FMR1* mRNA levels than both TDCs and FXS participants (Fig. 3a). This finding is consistent with *FMR1* mRNA levels in previous studies^22^. Next, we compared *FMR1* mRNA concentrations between males and females in each diagnostic category. There was no significant difference observed between the sexes in PMCs or TDCs; however, the sample size for male PMCs was very low making the interpretation of these results difficult. In participants with FXS, males had significantly lower *FMR1* mRNA levels than females, as expected (Fig. 3b).

**Figure 4:**
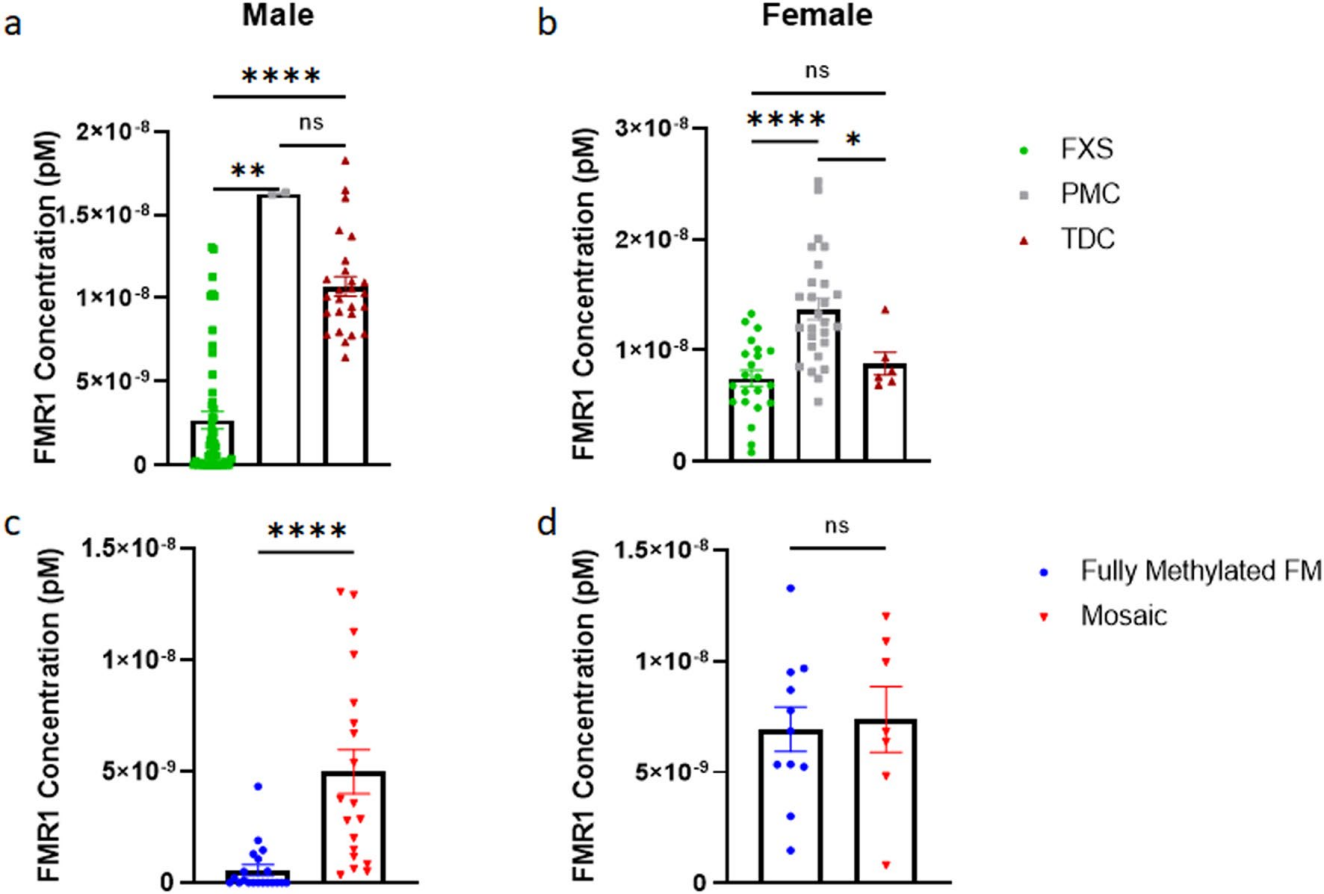
Males and females with FXS have reduced *FMR1* mRNA levels. **a** Males with FXS have significantly lower *FMR1* levels than male PMCs and TDCs (Kruskal–Wallis tests with the FDR method of Benjamini and Hochberg, *p* < 0.0001; *p*(FXS-PMC) = 0.0033, *p*(FXS-TDC) < 0.0001). There is no significant difference between male PMCs and male TDCs (*p* = 0.3404). **b** Female PMCs have significantly higher FMR1 mRNA concentrations than females with FXS and female TDCs (Kruskal–Wallis tests with the FDR method of Benjamini and Hochberg, *p* < 0.0001; *p*(PMC-FXS) < 0.0001, *p*(PMC-TDC) = 0.0264). There is no significant difference between FXS females and TDC females (*p* = 0.5612). **c** Fully methylated full mutation FXS males have significantly lower *FMR1* levels than FXS mosaic males (two-tailed Mann–Whitney test, U = 34, n_1_ = 20 n_2_ = 19, *p* < 0.0001). **d** In FXS females, there is no significant difference between fully methylated FM and mosaics (two-tailed Mann–Whitney test, U = 35, n_1_ = 11 n_2_ = 7, *p* = 0.7914). Error bars represent SEM. *FMR1* Fragile X Messenger Ribonucleoprotein 1, *FXS* Fragile X Syndrome, *PMC* Premutation Carriers, *TDC* Typically Developing Controls, *SEM* Standard Error of the Mean, *NS* Not Significant, ****Indicates *p* < 0.0001, **Indicates < 0.01, *Indicates < 0.05.

Next, we compared the diagnostic groups separated by sex. In males, *FMR1* mRNA levels were significantly lower in participants with FXS compared to PMCs and TDCs. There was no significant difference observed between male PMCs and TDCs (Fig. 4a); however, as mentioned above, the sample size for male PMCs was very low. In females, *FMR1* mRNA concentrations were significantly higher in PMCs compared to participants with FXS and TDCs. There was no significant difference observed between female participants with FXS and TDCs (Fig. 4b). Again, the sample size for female TDCs was low, and in the future, larger sample sizes should be analyzed.

Last, we compared FM–FM and mosaic FXS participants. The mosaic group was comprised of individuals with repeat and/or methylation mosaicism (see Supplemental Table 1 for subject level description of mosaicism available from Southern Blot and PCR testing). In males, we observed significantly lower *FMR1* levels in FM–FM compared to mosaic participants (Fig. 4c). In females, no significance was observed between the two groups (Fig. 4d).

### *FMR1* mRNA and FMRP levels in all participants are positively correlated

**Figure 5:**
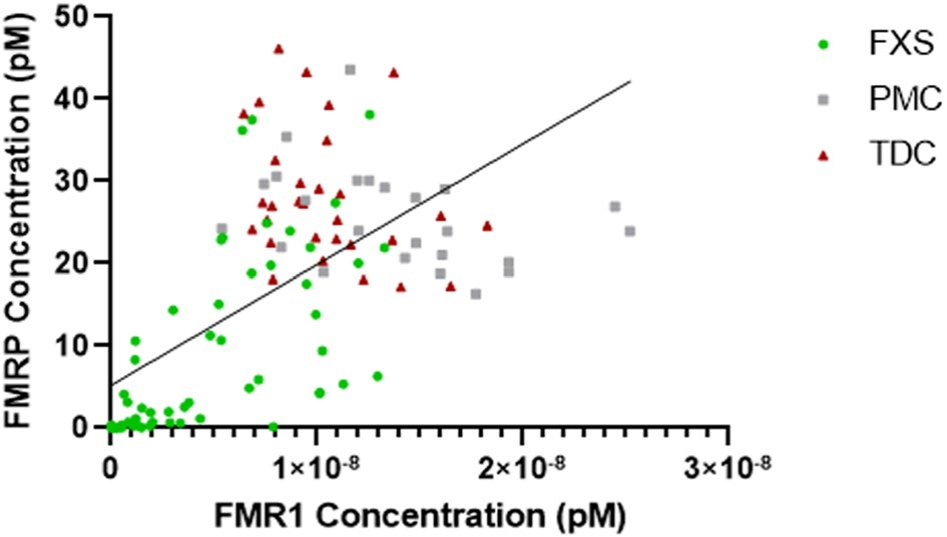
Linear correlation between *FMR1* mRNA and FMRP levels in all participants (Pearson’s correlation, r = 0.6390, R^2^ = 0.4084, *p* < 0.001). *FMR1* Fragile X Messenger Riboprotein 1, *FMRP* Fragile X Messenger Ribonucleoprotein.

Expression of the *FMR1* gene can be regulated on the transcriptional and translational level. We have previously reported an optimized, highly sensitive and reproducible assay to detect FMRP in dried blood spots^10^. To test the relationship between *FMR1* mRNA and FMRP levels, we analyzed the correlation of these levels from 120 participants. There was a linear correlation between *FMR1* mRNA and FMRP levels for all participants (Pearson’s correlation, r = 0.6390, R^2^ = 0.4084, *p* < 0.001) (Fig. 5).

### *FMR1* mRNA and FMRP levels are positively correlated in participants with a fully methylated FMR1 gene with > 200 CGG repeats

**Figure 6:**
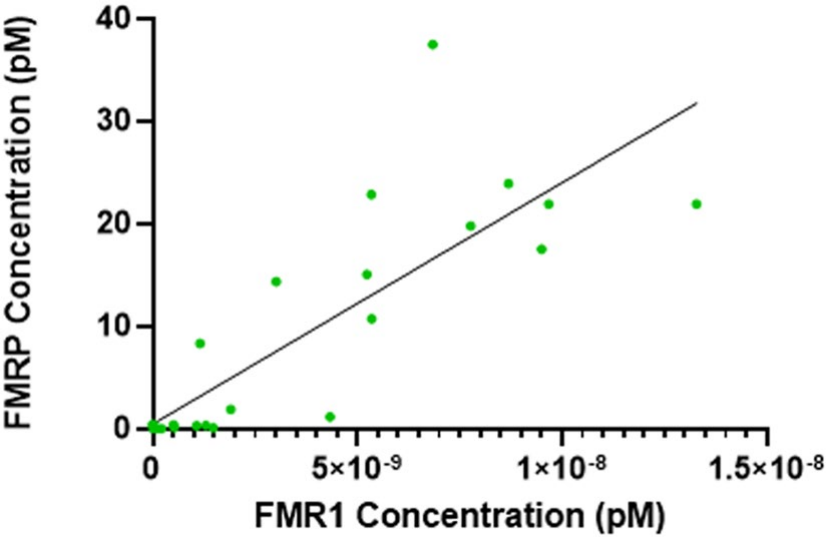
Linear correlation between *FMR1* mRNA and FMRP levels in fully methylated full-mutation (FM–FM) FXS participants (Pearson’s correlation r = 0.8479, R^2^ = 0.7190, *p* < 0.001). *FMR1* Fragile X Messenger Ribonucleoprotein 1, *FMRP* Fragile X Messenger Ribonucleoprotein.

We next analyzed the correlation between *FMR1* mRNA and protein levels in 30 FM–FM FXS participants for which both *FMR1* and FMRP levels were available. Four participants had multiple draws and their 20% trimmed mean values for *FMR1* mRNA and FMRP concentrations, respectively, were averaged. There was a positive linear correlation between *FMR1* and FMRP levels (Pearson’s correlation r = 0.8479, R^2^ = 0.7190, *p* < 0.001). When FMRP levels were outside the limit of detection (9 participants), the *FMR1* mRNA levels were within the limit of detection for 2 of the 9 participants but outside the limit of detection for the remaining 7, suggesting that *FMR1* mRNA is not always translated into protein. There were 3 participants with trace FMRP expression detected but their *FMR1* mRNA expression was outside of the limit of detection. This is likely due to the different sensitivities of the *FMR1* and FMRP assays (Fig. 6).

**Figure 7:**
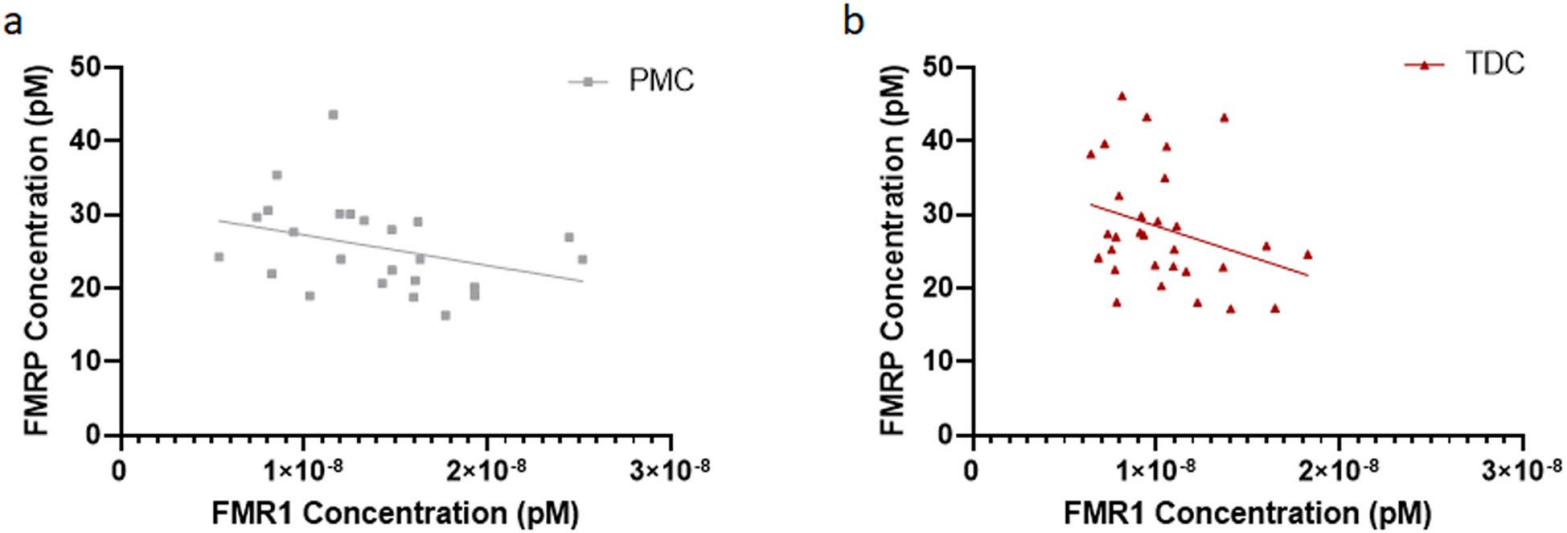
No significant linear correlation between *FMR1* mRNA and FMRP levels in blood from PMC participants (Pearson’s correlation, r = − 0.301, R^2^ = 0.0908, *p* = 0.1075) or TDC participants (Pearson’s correlation, r = − 0.343, R^2^ = 0.1175, *p* = 0.1011). *FMR1* Fragile X Messenger Riboprotein 1, *FMRP* Fragile X Messenger Ribonucleoprotein, *PMC* Premutation Carrier, *TDC* Typically Developing control.

By contrast, no significant correlation between FMRP and *FMR1* mRNA expression was detected when PMC (Fig. 7a) or TDC participants (Fig. 7b) were analyzed separately. Lack of a linear correlation between FMRP and *FMR1* expression in PMC and TDC participants is likely due to mechanisms inhibiting baseline *FMR1* translation when the mRNA is transcribed at or close to physiological levels^23^.

**Figure 8:**
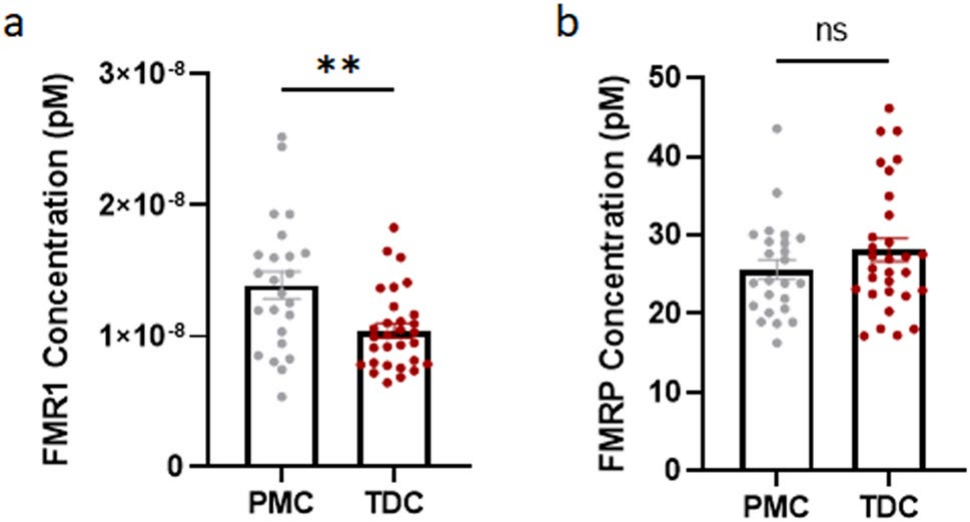
*FMR1* mRNA levels and FMRP levels in PMCs and TDCs. **a** *FMR1* mRNA levels are elevated in PMCs compared to TDCs (Mann–Whitney test, U = 200, n_1_ = 24 n_2_ = 30, *p* = 0.0048 two-tailed). **b** FMRP levels are not significantly different in PMCs and TDCs (Mann–Whitney test, U = 312, n_1_ = 24 n_2_ = 30, *p* = 0.4089 two-tailed). Male and female participants were combined. Error bars represent SEM. *FMR1* Fragile X Mental Retardation 1, *FMRP* Fragile X Messenger Ribonucleoprotein, *FXS* Fragile X Syndrome, *PMC* Premutation Carriers, *TDC* Typically Developing Controls, *SEM* Standard Error of the Mean, *ns* Not Significant, **Indicates *p* < 0.01.

When comparing PMCs with TDCs across sexes, elevated *FMR1* mRNA levels were observed in PMCs but there was no significant difference between PMC and TDC FMRP levels (Fig. 8a,b), again suggesting that FMRP synthesis is suppressed in PMCs due to the presence of the CGG repeat expansion in the 5′ untranslated region of the *FMR1* gene^23^. The relationship between CGG repeat count and mRNA, however, was not significant (r = 0.15, *p* = 0.46) for PMCs. Based on past literature, we also examined non-linear relationships; however, again found no significant relationship (*p* > 0.42).

### Increased *FMR1* mRNA levels are associated with higher IQ

**Figure 9:**
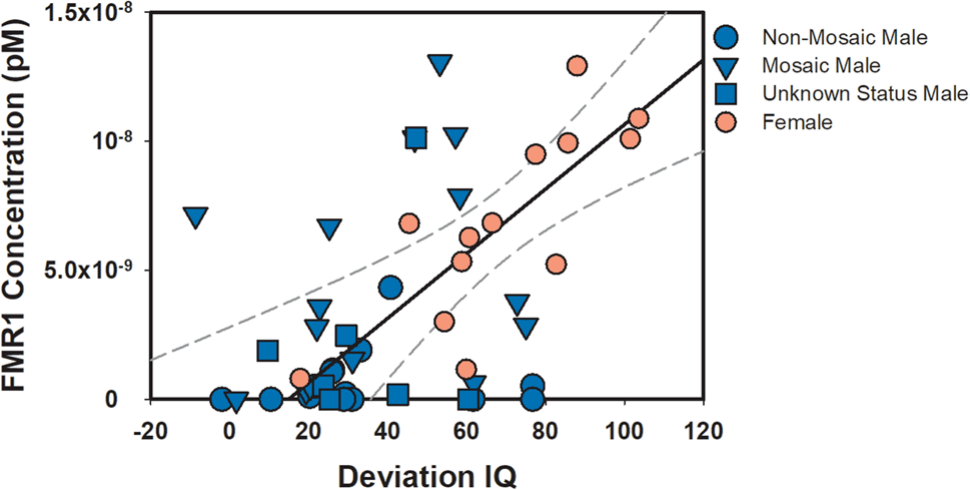
Linear correlation between *FMR1* mRNA and Deviation IQ across FXS participants (Pearson’s correlation r = 0.56, R^2^ = 0.31, *p* < 0.001). Males are depicted in blue based on mosaic status (circle = non-mosaic, triangle = mosaic male, square = mosaic status unknown due to unavailable PCR) and females are depicted in pink. Black solid line represents linear regression and dotted gray lines represent 95% confidence interval.

Across males and females with FXS, increased mRNA expression was associated with higher Deviation IQ scores (r = 0.56, R^2^ = 0.31, *p* < 0.001; Fig. 9). However, when examining sexes separately, the relationship between mRNA expression and IQ scores only was significant for females (r = 0.80, R^2^ = 0.64, *p* < 0.001), not males (r = 0.19, R^2^ = 0.04, *p* = 0.25). The difference between these correlation coefficients was significant (Z = 2.7, *p* = 0.01). Even after removing the male outlier, mRNA expression and IQ scores only trended towards significance in males with FXS (r = 0.29, R^2^ = 0.08, *p* = 0.09).

Next, we examined males with mosaicism and females together (n = 31), and we found a significant relationship between increased mRNA expression and higher IQ scores (r = 0.57, R^2^ = 0.32, *p* = 0.001). Then, we examined the relationship in males with > 0 mRNA expression and females (n = 43), and again found a significant relationship (r = 0.64, R^2^ = 0.41, *p* < 0.001). However, when we examined this subgroup of males alone, the relationship between *Fmr1* and IQ did not reach significance (r = 0.34, R^2^ = 0.11, *p* = 0.08). Still, our findings show mRNA expression accounts for over one-third of variance in IQ in individuals with FXS who have some mRNA expression.

## Discussion

This study reports a highly reproducible and sensitive qRT-PCR-based assay to detect *FMR1* mRNA in peripheral whole blood in typically developing controls and individuals with FXS. Our results suggest that even in individuals with CGG repeat expansions over 200 and hypermethylated *FMR1* gene (i.e., FM–FM males), low levels of *FMR1* mRNA can be reliably detected. Moreover, we show that trace amounts of *FMR1* mRNA are positively correlated with IQ in females but not in males. These results have two important implications. First, they confirm previous studies suggesting that in a subgroup of individuals meeting the current molecular diagnostic criteria of FXS, the *FMR1* gene is not completely silenced. Second, this trace expression of the *FMR1* gene can be physiologically relevant. In the future, determining *FMR1* expression in individuals with FXS could aid in their precise diagnosis, prediction of their disease trajectory, and potentially in identifying optimized treatment modalities.

A hallmark of FXS is that the phenotypic spectrum even within one diagnostic subcategory of FXS, e.g., fully methylated, fully mutated *FMR1* gene, can vary widely. Although our results are overall in line with the expectation that CGG expansion and hypermethylation lead to strongly reduced gene transcription, our results argue against the current categorical subdivision of individuals based on their repeat expansion (i.e., CGG repeat ≤ 44 normal; 44–54 intermediate or grey zone; 55–200 premutation, > 200 full mutation^2^). Instead, our findings suggest that the molecular diagnosis, similar to the phenotypic representation, reflects a continuous spectrum rather than discrete categories. This molecular spectrum may affect disease trajectory, as supported by our correlational analyses with IQ, and treatment response. Our findings indicate standard FXS diagnostic testing via Southern blot and PCR are not sensitive enough to account for the molecular variation identified here, highlighting the need to consider expanding current diagnostic practices to include more sensitive molecular assays.

Previous studies have used qRT-PCR to detect *FMR1* mRNA levels in blood from individuals with FXS^13,14,17,21^. Our results are a significant advance because we optimized sensitivity and rigor of the assay and established lower detection limits. Previous studies reported a surprising lack of individuals with zero *FMR1* gene expression^13^ or a large proportion of individuals with *FMR1*^17^. Here, we established a reliable lower limit of detection of *FMR1* gene expression based on rigorous testing and assay development, which will help to accurately interpret the results. Moreover, our detailed workflow based on a clearly defined decision tree will ensure reproducibility when repeated at different sites and in different laboratories, and thus could be the first step towards a diagnostic assay. Here, we analyzed a large number of participants (n = 138), which increases the confidence in the detected differences between diagnostic groups. Intra- and inter-assay variabilities were low (5 and 14%, respectively) and in line with the standard accepted variability for laboratory molecular assays (< 15%). Notably, inter-draw/inter-visit variability was higher suggesting that *FMR1* mRNA levels are sensitive to other factors that may vary between clinic visits, such as nutrition status, infections, and developmental status (i.e., age).

We detected a strong positive correlation between *FMR1* mRNA and FMRP levels, which was driven by FM–FM participants. This suggests that the transcribed mRNA in FM–FM participants is translated into protein and thus may have a positive functional impact as evidenced by a potential trending relationship with IQ scores among males who express > 0 mRNA. However, in a few cases, we detected *FMR1* mRNA with no or very little corresponding protein expression. In general, our data showed a broad range of FMRP protein levels for virtually the same amount of mRNA (shown as data points lined up on a vertical line), and it is thus unlikely that the observed variability is due to a certain threshold of *FMR1* mRNA that is required for FMRP production. Interestingly, the occurrence of AGG triplets in the CGG repeat expansion region does not seem to influence *FMR1* mRNA translation^24^. We speculate that other epigenetic factors play a role such as regulation by *FMR1-*targeting mRNA binding proteins and microRNAs^25,26^. Moreover, insufficient translation of the mRNA transcribed from a CGG-expanded gene, could be due to extensive secondary structures in the 5’UTR of the expanded gene impeding ribosomal scanning or repeat-associated non-AUG-initiated translation of expanded CGG repeats (CGG RAN)^23^. In line with this hypothesis, there was no significant linear correlation between *FMR1* mRNA and FMRP levels in PMC or TDC participants, suggesting that, at physiological levels, *FMR1* mRNA translation in peripheral blood is regulated tightly, similarly as observed in neurons^23^. Our findings that *FMR1* mRNA levels in PMCs are increased compared to typically developing controls, despite FMRP levels not being significantly different between the two groups, further confirms that the CGG repeat expansion reduces translation efficiency. Of note, although overall expressing more *FMR1* mRNA than TDC participants, PM carriers showed a wide range of mRNA levels, including individuals with lower than normal levels. Size and methylation mosaicism has been shown in premutation carriers^27^, and in rare occasions, premutation alleles can by hypermethylated^28^, which could contribute to these low expressing individuals. In the future, it will be important to determine both *FMR1* mRNA and protein levels across larger sample sizes and different conditions to fully understand the molecular phenotype.

Our studies suggest that there are no significant sex differences in *FMR1* mRNA levels between either premutation carriers or typically developing controls, and that *FMR1* mRNA levels are stable over time. However, interpretability of these results is limited due to small sample sizes (especially in the group of male premutation carriers) and lack of systematic analysis of *FMR1* mRNA levels between age groups. Future studies are needed to better understand the effects of sex as well as age and development on *FMR1* gene expression.

The molecular mechanisms leading to low level *FMR1* mRNA expression in individuals diagnosed with FXS are currently unknown. As alluded to above, we speculate that “leaky expression” despite a > 200 CGG repeat expansion and hypermethylation of the gene, in addition to other genetic and environmental modifiers could contribute. The diagnosis of FXS currently relies on genetic testing for repeat expansion and hypermethylation based on PCR and Southern Blotting^29^. These methods in general have a low sensitivity^30^, and thus subtle differences in CGG repeat length and methylation remain undetected. Our present and previously published results showing that hypermethylated individuals with FXS with a CGG repeat expansion > 200 express *FMR1* mRNA and protein which affects their IQ illustrates the need to develop and use more sensitive methods to analyze the *FMR1* gene. Combined with quantification of *FMR1* mRNA and protein analysis, such a method could provide a more accurate diagnosis of FXS.

Correlational findings support the clinical utility of our *FMR1* mRNA assay by demonstrating the relationship between increased mRNA expression and higher IQ, as reported previously^13^. It is important to note that this relationship was driven by females with FXS, consistent with our correlational findings with FMRP and IQ and previous studies showing a relationship between FMRP expression and neurobehavioral function in females^9,10^. Although males with FXS who had > 0 mRNA expression seemed to follow this trend, the relationship with IQ did not reach significance. Our findings implicate mRNA has a “dose-dependent” effect on disease severity, especially in females with FXS, with increased mRNA expression corresponding to a more subtle clinical phenotype and reduced mRNA expression corresponding to a more severely impacted phenotype. This is in contrast to a recent study by Baker et al., (2020) who found higher mRNA levels were associated with increased irritability in full mutation males with incomplete silencing^21^. Authors argued potential toxic gain to function role of mRNA in this subgroup, though different methods of mRNA quantification also may account for these findings.

Notably, among males with FXS with some *FMR1* expression, our findings suggest that predicting functional outcomes may be less precise, though we still may be able to provide broad estimates which can be clinically meaningful to professionals and families. Similarly, our findings among males with no *FMR1* expression, who demonstrate a wide range of Deviation IQ scores (− 2 to 76), also highlight that additional factors, such as genetic modifiers or socioeconomic and environmental factors, play a role in FXS disease expression. Although our findings among males may be partially explained by the artificial detection limits of the assay, it is more likely genetic modifiers and social determinants of health account for variance in IQ. In the future, to fully understand the FXS disease phenotype and to best treat affected individuals, these factors will have to be analyzed in detail.

Our study is not without limitations. For example, a limitation of our study is that the lower limits of detection are determined by the technical constraints of the assays used, i.e. *FMR1* qRT-PCR or Luminex-based FMRP assays. It is thus likely that some of the samples currently determined as zero mRNA and protein, may, in fact, express *FMR1* mRNA and/or protein, which could affect their disease trajectory and treatment response. Despite the relatively large patient sample size across a large age range, we were not able to assess *FMR1* levels across development. In future studies, it will be imperative to examine mRNA and protein levels longitudinally in younger aged individuals with FXS, especially in light of the higher variability we observed between draws and across visits, which, in some cases were more than a year apart from each other. It is presently unknown whether *FMR1* expression changes during development in humans but will be important to determine for both individualized treatment planning and potential gene-modifying therapeutics in the future. Last, the clinical data presented is limited to IQ, thus future studies are needed to better understand the relationship between *FMR1* expression and clinical outcomes using multimodal, highly quantitative phenotyping approaches. It also will be important to further examine relationships in PMC to determine the potential effects of toxic mRNA levels that has been implicated in a recent study^21^.

In summary, here, we report a sensitive, quantitative, and reproducible qRT-PCR assay to reliably detect *FMR1* mRNA in whole blood. Our results show that individuals with FXS that have a CGG repeat length of over 200 can express low levels of *FMR1* mRNA, which may lead to FMRP expression and altered cognitive function. Future studies are needed to understand these relationships and leverage our findings to improve clinical care.

## Methods

### Participants

A total of 138 participants aged 1–67 years were enrolled in this study (Table 1). The Cincinnati Fragile X Research and Treatment Center recruited participants with Fragile X Syndrome (FXS) (55 male, 22 female), premutation carriers (PMCs) (2 male, 27 female), and typically developing controls (TDCs) (26 male, 6 female). Rush University completed clinical southern blot (SB) and/or polymerase chain reaction (PCR) testing on 105 participants (76 with FXS, 26 PMCs, and 3 TDCs) to confirm diagnosis and to evaluate repeat and/or methylation mosaicism status*.* Only one out of 77 FXS participants did not have clinical testing at the time of this study. In these tests, 26 of the 76 participants with FXS were classified as repeat and/or methylation mosaics. TDCs were recruited through online advertisement and did not have a history of developmental or neuropsychiatric disorders. All participants or legal guardians gave written or verbal assent. The CCHMC Institutional Review Board approved this project. Human subject work followed all relevant regulations and was in accordance with the Declaration of Helsinki.

### Blood collection and processing

Blood was collected from each participant in a PaxGene® Blood RNA Tube (#762165, Qiagen, Hilden, Germany), which was inverted 10 times before being stored at − 20 °C or − 80 °C. PaxGene tubes are rated stable for at least 11 years when stored between − 20 °C and − 70 °C. All tubes used for this study were stored for less than 3 years before the RNA was extracted.

RNA was extracted using the PaxGene® Blood RNA Kit (#762164, PreAnalytiX GmbH, Hombrechtikon, Switzerland) according to the manufacturer’s protocol. Approximately 80 µL of RNA was extracted and 2 µL of each sample were analyzed in duplicates on the Take3™ Micro-Volume Plate (BioTek Instruments Inc., Winooski, Vermont, U.S.) using a Cytation™ 3 Cell Imaging Multi-Mode Reader (BioTek Instruments Inc., Winooski, Vermont, U.S.). Gen5™ Image + software (BioTek Instruments Inc.) to determine the concentration and the ratio of absorbances at 260 and 280 nm. The average 260/280 ratio of the samples was 2.087 ± 0.019 indicating acceptable quality. To minimize the number of freeze-thaws, RNA was aliquoted into 10 µL aliquots. RNA aliquots were stored at − 80 °C.

The first 16 extracted RNA samples were not aliquoted until after the first freeze–thaw. The next 11 samples were aliquoted, frozen, and then thawed to change labels. These 27 samples were thawed twice before their final analysis. The rest of the samples were only thawed once. The typically developing human control samples that were used on every plate (TDC #1) to assess variability were thawed 1–3 times before they were aliquoted. We conducted an experiment to evaluate the effect of 1–5 freeze–thaw cycles on extracted RNA. Tubes were removed from − 80 °C storage, thawed on ice for approximately 10 min, and then stored in the freezer for 30 min. This was repeated until a predetermined number of freeze–thaw cycles for each sample had been completed. Two tubes were used for each condition. Based on our results, there was no significant difference between 1 and 5 freeze–thaw cycles (shown in Fig. 1, and further described in the “Results” Section).

### cDNA synthesis

cDNA was generated from mRNA using the High-Capacity RNA-to-cDNA™ Kit (#4388950, Thermo Fisher Scientific, Waltham, Massachusetts, U.S.) following the manufacturer’s protocol. In each reaction, 0.3 µg of RNA was used unless the RNA concentration was too low. In this case, 0.2 µg (1 sample) or 0.1 µg (4 samples), was used depending on the concentration. Our analyses showed that the cDNA and following quantitative real-time PCR (qRT-PCR) reactions were quantitative within a range of 0.1 to 1 μg (linear regression analyses yielded R^2^ values > 0.99 two independent experiments using 1, 0.6, 0.3, 0.2, 0.1 and 0.075 μg of RNA input, *data not shown*). After completion of the reaction, the cDNA was either stored at 4 °C for less than a week or was immediately used for qRT-PCR analysis. After a sample had an acceptable qRT-PCR result or the cDNA had been stored at 4 °C for a week, the sample was not analyzed again. If the sample's Ct value was above the last standard point’s Ct value and the RNA concentration was high enough, 0.6 µg (6 samples) or 1 µg (5 samples) of RNA was used for the qRT-PCR assay and *FMR1* mRNA values were adjusted to reflect the content in 0.3 μg (Fig. 2).

### Quantitative real-time PCR (qRT-PCR) Analysis

Quantitative real time PCR (qRT-PCR) was used to determine the amount of *FMR1* mRNA in a sample. A standard curve prepared from a plasmid containing the human *FMR1* open reading frame was run with each assay (“plate”) to allow absolute quantification of *FMR1* mRNA in 0.3 μg of mRNA from whole blood. Over many assays, we determined the conditions that produced the most reliable and reproducible results. As part of the optimized conditions, the standard curve dilutions were always prepared on the day of the qRT-PCR reactions and the cDNA was stored at 4 °C for less than a week.

### Standard curve preparation

A 9-point standard curve based on a 1:5 dilution series was prepared with AAV-CAG-*FMR1* plasmid (6598 base pairs) cloned by the Gross lab, based on Addgene plasmid #28014 and *FMR1*(NM_002024) ORF Clone (GeneScript®, #Ohu21442). AAV-CAG-GFP was a gift from Karel Svoboda (Addgene plasmid # 28014; RRID:Addgene_28014)^31^. The plasmid was diluted to 1 ng/µL (2.3E−4 pM) and stored in 6 and 16 μL aliquots at − 20 °C. The standard curve covered a range of 4.6E−5 to 1.776E−10 pM *FMR1* mRNA. The lowest standard curve point was determined by testing a range of concentrations to identify the lowest concentration that reliably detected signal above the no template control.

### Primer design

Primers were designed using the “Universal Probe Library Assay Design Center” (Roche LifeScience, Basel, Switzerland) and synthesized by Thermo Scientific (Thermo Fisher Scientific, Waltham, Massachusetts, U.S.) to amplify a sequence in the coding region of the human *FMR1* gene (nucleotides 547-628 of FMR1, transcript variant ISO1, NM_002024.6). Primer sequences were as follows:

Forward = 5′—TAT GCA GCA TGT GAT GCA ACT—3′.

Reverse = 5′—TTG TGG CAG GTT TGT TGG GAT—3′.

Primers were reconstituted to 100 µM solutions using purified water (ddH_2_O), and then diluted to 20 µM working solutions. These primers produced single melting curve peaks at ~ 76 °C.

### qRT-PCR assay

The qRT-PCR was performed in 20 µL reactions using the iTaq Universal SYBR® Green Supermix (#1725121, BIO-RAD, Hercules, California, USA) following the manufacturer’s protocol. To avoid pipetting errors, one master mix per plate was prepared that contained the iTaq Universal SYBR® Green Supermix (10 µL/reaction), primers (0.15 μL/reaction, 150 nM each) and ddH_2_O up to a volume of 19 μL per reaction. Note that our optimization experiments identified a primer concentration of 150 nM as optimal, which is below the 300–500 nM recommendation of the manufacturer. 19 μL of the master mix followed by 1 μL of the sample per reaction were loaded into MicroAmp™ Fast Optical 96-Well Reaction Plates (#4346906, Thermo Fisher Scientific, Waltham, Massachusetts, U.S.). The standard curve and no template control (NTC) (run on each plate, substituting sample with ddH_2_O) were loaded in triplicates, while participant’s samples were loaded in quintuplicates. On each plate, cDNA from the same typically developing human control participant (TDC #1) was loaded to measure assay variability. After samples were loaded, the plate was sealed with a MicroAmp™ Optical Adhesive Film (#4311971, Thermo Fisher Scientific, Waltham, Massachusetts, USA) and then centrifuged at 1500 RPM for 5–10 min at room temperature. The qRT-PCR reaction was run on the QuantStudio™ 3 Real-Time PCR System (Thermo Fisher Scientific, Waltham, Massachusetts, USA). The thermal cycling conditions include a hold stage (50 °C for 2 min then 95 °C for 10 min), PCR stage (95 °C for 15 s then 60 °C for 1 min for 40 cycles), and a melt curve stage (95 °C for 15 s, 60 °C for 1 min, 95 °C for 15 s).

### *FMR1* quantification

qRT-PCR analysis and *FMR1* quantification were done with the QuantStudio Design & Analysis Software v1.5.1 (Thermo Fisher Scientific, Waltham, Massachusetts, U.S.) using the automatic baseline threshold algorithm combined with the standard curve method. The standard curve was plotted with cycle threshold (Ct) on the y-axis and the quantity of each standard point on the x-axis. Quantities of the samples were interpolated from their Ct values using the standard curve.

### Quality control

Several criteria were established to determine if an assay was acceptable. First, all standard curve points and samples had to have a single melting curve peak at the expected melting temperature (~ 76 °C), and no or negligible amplification in the no template control. Then the slope, efficiency percentage, and correlation coefficient (R^2^) of the standard curve were calculated by the software. Based on these measurements and qRT-PCR criteria described in the literature^32^, we created three criteria for the standard curve, and thus a qRT-PCR plate, to be accepted: (1) Each standard point must have at least two replicates that have a Ct standard deviation below 0.5 and pass the Grubb’s outlier test. If the Ct standard deviation is greater than 0.5 for a set of triplicates, the analysis software will flag the set of triplicates. If a single replicate in the triplicate does not pass the Grubb’s test, the analysis software will flag that well. In both scenarios, we evaluate the replicate group, visually identify the well that is highly divergent from the two others on the amplification plot and remove it from analysis. (2) The efficiency must be between 90 and 100%. General guidelines recommend an efficiency between 90 and 110%. In our experiments assessing inter-assay variability, we calculated a CV of 29.13% when the efficiency of a plate was between 90 and 110%. When the efficiency was between 90 and 100%, the inter-assay variability CV was 14.20%. Therefore, we determined an efficiency of 90–100% was optimal for the reproducibility of this assay. (3) The R^2^ value must be greater than 0.980. Compiled results for these criteria are shown in Table 2 and are further discussed in the “Results” Section. Once the plate was accepted, each sample went through a decision tree (Fig. 2).

### qRT-PCR sample decision tree

The decision tree is illustrated in Fig. 2, and briefly described here. If a sample had a Ct value above the last standard point’s average Ct, the amount of RNA used in the cDNA reaction for this qRT-PCR plate determined the next step. For the samples with less than 0.3 µg due to an insufficient RNA concentration, we could not definitively state that the high Ct was due to an insufficient RNA concentration or if it was due to the participant not expressing the *FMR1* gene. These samples were removed from analysis (4/148). For the participants for which 0.3 µg was used in the cDNA reaction, their RNA concentration determined the next step. If a sample did not have a sufficient RNA concentration to use greater than 0.3 µg in the cDNA reaction, their *FMR1* concentration was set to 0 (5/148). If a sample had a sufficient RNA concentration for 0.6 or 1.0 µg to be used in the cDNA reaction, the qRT-PCR assay was repeated with cDNA prepared from 0.6 or 1.0 μg. If the sample’s Ct was still above the last standard point’s Ct after this adjustment, their *FMR1* concentrations were set to 0. 10 out of 11 samples that were ran with 0.6 or 1.0 μg had their *FMR1* concentrations set to 0. For the samples that had Ct values below the last standard point’s Ct, they had to pass the following criteria: Five replicates with (1) a CV < 15%, (2) no outliers as determined by the software, and (3) amplification in all five wells. If the sample did not pass the criteria, the qRT-PCR assay was repeated for this sample. If the sample did pass the criteria (129/148 samples), the participant’s *FMR1* values were normalized to 0.3 µg and their 20% trimmed mean values (average of the 3 central values after removing the lowest and highest value) were used to represent the patient’s *FMR1* expression. For participants with multiple draws (6 participants with 2 draws, 2 participants with 3 draws) or two accepted runs with the same RNA tube (14 participants), their 20% trimmed mean values were averaged and represented their *FMR1* concentration. For one of the participants with two draws, there were two accepted runs with the same RNA tube for the first draw and one for the second draw. The average of the 20% trimmed mean values for all three assays was used to represent this participants *FMR1* concentration.

### Assay reproducibility

The reproducibility of this assay was determined by intra-assay, inter-assay, and inter-draw variability. The intra-assay variability, or the variability between replicates on one plate, was calculated by averaging the CV for each set of quintuplicates. Results are reported in Table 3, and procedures described in detail below.

The inter-assay variability, or the variability between different plates, was calculated with TDC #1 and 14 other participants. RNA from three different RNA tubes drawn at the first visit of TDC #1 were used to prepare cDNA, which was used on all 27 plates in this study. The inter-assay variability for TDC #1 was determined by calculating the CV of the 20% trimmed mean for one of the three RNA tubes, ran across 15 plates. For the 14 participants, the inter-assay variability was determined by averaging the CV between each participant’s 20% trimmed means from two different plates. These 14 participants were chosen to span a wide variety of *FMR1* quantities and had the following diagnostic backgrounds: 3 fully methylated FM FXS males with *FMR1* mRNA values and FMRP concentrations set to 0, 1 fully methylated FM FXS male with trace FMRP concentrations, 2 fully methylated FM FXS females, 2 FXS mosaic males (1 repeat mosaic, 1 repeat and methylation mosaic), 2 FXS mosaic females (1 repeat mosaic, 1 repeat and methylation mosaic), 1 male TDC, 1 female TDC, and 2 female PMCs.

Inter-draw variability, or variability between different draws, was calculated for tubes drawn on the same day and for tubes drawn on different days. The inter-draw variability on the same day was determined by the CV of the 20% trimmed mean values from three RNA tubes provided by TDC #1 on visit 1. The inter-draw variability on different days was determined by the CV of the 20% trimmed mean values of 2 or more different tubes drawn on separate days from TDC #1 and eight additional patients. Samples from TDC #1 and the eight participants with multiple draws were used separately for this analysis.

### Initial clinical utility correlational analysis

In order to initially establish the clinical utility of the mRNA assay, we examined the linear relationship between peripheral mRNA expression and intellectual functioning. We examined a subset of participants with FXS (n = 53, 72% male, age range 4–61 years, mean age 20.4 ± 14.2) who completed the Abbreviated Battery of the Stanford-Binet, Fifth Edition (SB-5) and used their deviation IQ scores to provide best estimate of intellectual ability^33^ as previously done^10^. To determine the relationship between mRNA expression and IQ, we conducted Pearson correlations with SPSS 19.0. One mosaic male was identified as an outlier based on visual inspection and confirmed as falling outside the 95% confidence interval of the linear regression.

### FMRP assay

FMRP was quantified from blood spots using an optimized Luminex assay as described in Boggs et al^10^. Parts of the FMRP data used to assess correlations of *FMR1* mRNA with FMRP were reported in Boggs et al.^10^.

### Statistical analyses

For statistical analysis of *FMR1* mRNA expression, GraphPad Prism 9.3.1 and SPSS 19.0 software were used. When comparing the *FMR1* mRNA expression between different groups, the following tests were used depending on experiment design: Kruskal–Wallis tests with the original FDR (false discovery rate) method of Benjamini and Hochberg or two-tailed Mann–Whitney tests. Significance was determined as a *p* value < 0.05.

### Ethics approval and consent to participate

The Cincinnati Children’s Hospital Medical Center Institutional Review Board (IRB # 2013-7327) approved all human experiments in this study. All participants provided informed consent themselves or participants under guardianship (minor or adults) had their legal guardian provide consent for participation in this study. Participants 12 years of age and older provided assent, when possible.

## Supplementary Information

Supplementary Information.

## Data Availability

Data are shown in tables and graphs in the manuscript. Raw data are available upon reasonable request.
