# Correction: A sensitive and reproducible qRT-PCR assay detects physiological relevant trace levels of *FMR1* mRNA in individuals with Fragile X syndrome

**DOI:** 10.1038/s41598-025-28355-1

**Published:** 2025-12-12

**Authors:** Devan Straub, Lauren M. Schmitt, Anna E. Boggs, Paul S. Horn, Kelli C. Dominick, Christina Gross, Craig A. Erickson

**Affiliations:** 1 Division of Child and Adolescent Psychiatry, Cincinnati Children’s Hospital Medical Center, 3333 Burnet Ave., Cincinnati, OH 45229-3039 USA; 2 Division of Behavioral Medicine and Clinical Psychology, Cincinnati Children’s Hospital Medical Center, 3333 Burnet Ave., Cincinnati, OH 45229-3039 USA; 3 Department of Pediatrics, University of Cincinnati College of Medicine, 3333 Burnet Ave., Cincinnati, OH 45229-3039 USA; 4 Department of Psychiatry and Behavioral Neuroscience, University of Cincinnati College of Medicine, Stetson Building Suite 3200, 260 Stetson Street, Cincinnati, OH 45267-0559 USA; 5 Division of Neurology, Cincinnati Children’s Hospital Medical Center, 3333 Burnet Ave, Cincinnati, OH 45229-3039 USA

Correction to: *Scientific Reports* 10.1038/s41598-023-29786-4, published online 07 March 2023

The original version of this Article contained an error in Table 1, where the value for “Typically developing controls” in the column “Range” for the *FMR1* concentration was incorrect. The correct and incorrect values appear below.

Incorrect:

**Table:** 

	*FMR1* concentration (pM)
	Range
Typically developing controls	1.62E-08 to 1.63E-08

Correct:

**Table:** 

	*FMR1* concentration (pM)
	Range
Typically developing controls	6.43E-09 to 1.83E-08

The original Article has been corrected.

