# Supplementary material for: A sensitive and reproducible qRT-PCR assay detects physiological relevant trace levels of FMR1 mRNA in individuals with Fragile X syndrome
Source: Sci Rep. 2023 Mar 7;13:3808. doi: 10.1038/s41598-023-29786-4 (PMC9992378; doi:10.1038/s41598-023-29786-4)
Supplement: Supplementary file 1 — Supplementary Information. [file 41598_2023_29786_MOESM1_ESM.pdf]

A sensitive and reproducible qRT-PCR assay detects physiological relevant trace levels of *FMR1* mRNA in individuals with Fragile X Syndrome

Devan Straub[1], Lauren M. Schmitt[2,3], Anna E. Boggs[1], Paul S. Horn[3,5], Kelli C. Dominick[1,4], Christina Gross[3,5], and Craig A. Erickson[1,4*]

Supplemental Table 1. Detailed Mosaicism Data for Participants with Size and/or Methylation Mosaicism

Subject	Sex	Size	Methylation
1	M	129, >200 repeats	10 kb and additional bands of 3 and 3.2 kb; Premutation is unmethylated
2	M	47, >200 repeats	
3	M	200, > 200 repeats	
4	M	200, > 200 repeats	
5	F	40 and 128, 162, and >200 repeats	
6	M	76, >200 repeats	
7	F	30, 165 and >200 repeats	5.2 kb and 2.8 kb and additional bands of 5.7 and a smear of 8-9 kb
8	F		5.2 kb and 2.8 kb and additional bands of 6-6.5 kb and 3.6 kb
9	F	30, >200 repeats	
10	M		Fragile X amplification mutation in the form of a fully methylated full mutation, and with mosaicism for an allele that is not detected on PCR and thus probably represents a deleted allele from the expanded allele with the primer site deleted
11	F	30, 134 and >200 repeats	5.2 kb and 2.8 kb and a smear of 3.1, 3.3 and 3.6 kb; UM FM, Premutation is methylated on the inactive X chromosome
12	M	46, 95 and >200 repeats	6, 7 and 8 kb and additional band of 2.9 kb; FM FM, Low level premutation and gray zone mosaicism
13	F	30, 118 and >200 repeats	5.2 kb and 2.8 kb and additional smear of 3.2 kb to 3.5 kb
14	M	71, 129 and >200 repeats	6-10 kb and additional bands of 3 and 3.2 kb
15	M	28, >200 repeats	
16	F	22, 149, and >200 repeats	
17	M	51, >200 repeats	
18	M	100, >200 repeats	8 kb and an additional light band of 2.9 kb; fragile X amplification mutation in the form of a fully methylated full mutation with mosaicism for a premutation
19	M	133, 184, and >200 repeats	6 kb and 3.3-3.4 kb; fragile X amplification mutation in the form of a fully methylated full mutation with mosaicism for a premutation. The premutation is unmethylated

20	M	168, 177, and >200 repeats	5.8-7.5 kb and a band of 3.3 kb; fragile X amplification mutation in the form of a fully methylated full mutation with mosaicism for a premutation
21	M	120, 186, and >200 repeats	
22	M	199, >200	
23	M	43, >200 repeats	
24	M	100, >200 repeats	8-8.5 kb and bands of 6 kb, 3.3 kb, 3 kb; fragile X amplification mutation in the form of a fully methylated full mutation with mosaicism for a premutatiom. The premutation is unmethylated
25	M	76, >200 repeats	
26	M	59, >200 repeats	
27	M	65, >200 repeats	
28	M	30, 195, and >200 repeats	
29	M	180, >200 repeats	
